# Histone H3E73Q and H4E53A mutations cause recombinogenic DNA damage

**DOI:** 10.15698/mic2020.07.723

**Published:** 2020-04-24

**Authors:** Pedro Ortega, Desiré García-Pichardo, Marta San Martin-Alonso, Ana G. Rondón, Belén Gómez-González, Andrés Aguilera

**Affiliations:** 1 Centro Andaluz de Biología Molecular y Medicina Regenerativa (CABIMER), Universidad de Sevilla-CSIC-Universidad Pablo de Olavide, Seville, Spain.

**Keywords:** chromatin, DNA replication, recombination, histone mutants

## Abstract

The stability and function of eukaryotic genomes is closely linked to histones and to chromatin structure. The state of the chromatin not only affects the probability of DNA to undergo damage but also DNA repair. DNA damage can result in genetic alterations and subsequent development of cancer and other genetic diseases. Here, we identified two mutations in conserved residues of histone H3 and histone H4 (H3E73Q and H4E53A) that increase recombinogenic DNA damage. Our results suggest that the accumulation of DNA damage in these histone mutants is largely independent on transcription and might arise as a consequence of problems occurring during DNA replication. This study uncovers the relevance of H3E73 and H4E53 residues in the protection of genome integrity.

## INTRODUCTION

Genetic instability is prevented through multiple processes to avoid cell death and tumorigenesis. Mechanisms ensuring replication fidelity as well as DNA damage checkpoints and repair pathways have evolved as a way to preserve genome integrity [1]. Accumulated evidence supports that most genetic instability sources arise from unrepaired DNA damage, such as double strand breaks (DSBs), or failures during DNA replication that can also ultimately lead to breaks. In this context, transcription is an important cause of replication stress, by directly or indirectly triggering transcription-replication conflicts [2].

The complex DNA organization into chromatin via packaging with histone and non-histone proteins influences all of these processes occurring at the DNA [3]. Chromatin structure exerts a major spatiotemporal control of DNA replication, repair and transcription processes thus affecting both the generation of endogenous damage as well as its efficient repair. Hence, whereas different chromatin states can favor or impede DNA damage occurrence by enhancing or diminishing the accessibility of genotoxic agents, chromatin relaxation upon DNA damage promotes access of the repair machinery to the DNA lesion [4]. Moreover, chromatin can play an active role in regulating DNA repair, as first exemplified by the phosphorylation of the serine 189 of mammalian H2AX histone variant (serine 129 phosphorylation of H2A in yeast, P-H2A), one of the earliest signals of the DNA damage checkpoint that expands up to 2 Mb around DSBs initiating a cascade of recruitment of repair factors [5]. Since this modification was discovered, several other histone posttranslational modifications have been described to affect DNA damage repair, including the methylation of lysine and arginine, phosphorylation of serine and threonine and acetylation, ubiquitylation or sumoylation of lysine [6].

To explore the possible role of histones H3 and H4 residues in the maintenance of genome integrity in a systematic manner, we took advantage of a hyper-recombination screening performed in a library of non-essential histone H3 and H4 mutants of *Saccharomyces cerevisiae* [7]. Here we describe two mutations in histones H3 and H4 (H3E73Q and H4E53A) that increase the levels of spontaneous recombinogenic DNA damage. Our results suggest that damage accumulates as a consequence of problems during DNA replication, supporting a role of these histone residues in the maintenance of genome integrity by ensuring proper replication.

## RESULTS

### H3E73Q and H4E53A mutations increase the spontaneous levels of direct-repeat recombination

**Figure 1:**
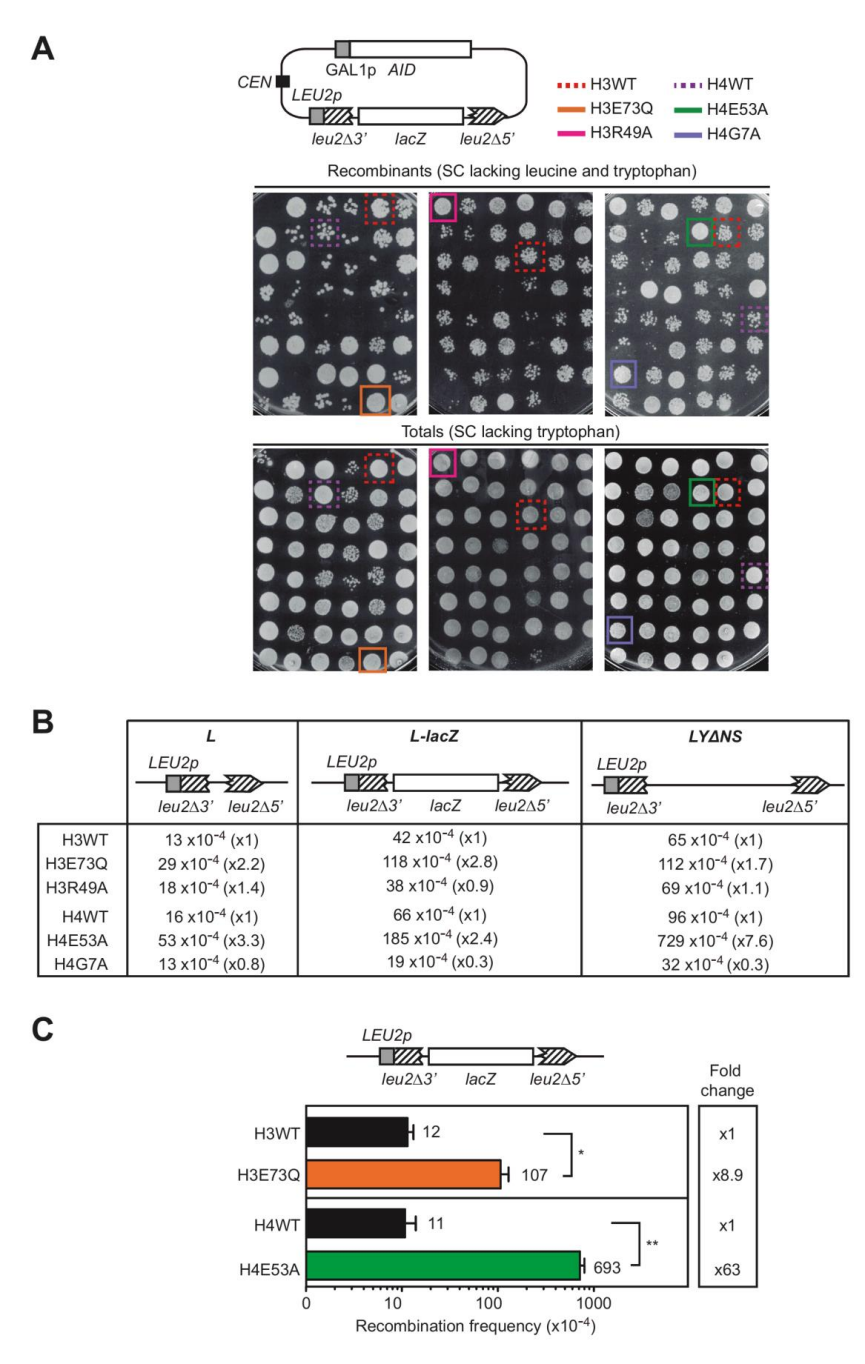
FIGURE 1: Histone H3E73Q and H4E53A mutants cause a hyper-recombination phenotype. **(A)** A scheme of the pLZGAID plasmid is shown. Visual analysis of direct-repeat recombination frequencies after AID overexpression in WT and histone mutant strains from the collection [8] transformed with pLZGAID. Similar dilutions of cultures grown in galactose media in 96-well-plates were plated in SC lacking leucine and tryptophan to detect Leu+ colonies (Recombinants) and in SC lacking tryptophan to visualize the total cells (Totals) and incubated for 3 days. Wild-type (H3WT), H3E73Q (H3E73Q)*,* H3R49A (H3R49A), H4 wild-type (H4WT), H4E53A (H4E53A) and H4G7A (H4G7A) strains are pointed out. **(B)** A scheme of the L, L-*lacZ,* and LYΔNS direct-repeat recombination system is shown. Analysis of median direct-repeat recombination frequencies in random colonies from H3 wild-type (H3WT), H3E73Q (H3E73Q)*,* H3R49A (H3R49A), H4 wild-type (H4WT), H4E53A (H4E53A) and H4G7A (H4G7A) strains transformed with pRS316-L, pSCH204, and pRS316-LYΔNS respectively. **(C)** A scheme of the L-*lacZ* direct-repeat recombination system is shown. Analysis of direct-repeat recombination frequencies in H3 wild-type (H3WT), H3E73Q (H3E73Q)*,* H4 wild-type (H4WT) and H4E53A (H4E53A) strains transformed with pSCH204 (n = 3). Means and SEM are plotted. *p ≤ 0.05, **p ≤ 0.01 (two-tailed Student's t-test).

To study the relevance of particular histone residues in genetic stability we took advantage of a previously performed screening that analyzed the recombination frequency using a direct-repeat recombination system in a collection of non-essential histone H3 and H4 mutants in *S. cerevisiae,* in which one of the loci encoding for histone H3 and H4 genes (*hht1-hhf1)* was deleted*,* and the other one (*hht2-hhf2)* was replaced by a mutant copy [7]. This library contains 423 alleles that included each of the H3 and H4 residues substituted by alanine, original alanines substituted by serine as well as different substitutions of all modifiable residues by amino acids mimicking modified and unmodified states and sets of systematic deletions of the histone N-terminal tails [8]. The screening was originally performed to identify histone residues that protect cells from accumulating DNA:RNA hybrids by selecting the mutations that enhanced recombination between direct repeats after the overexpression of AID (Activation-Induced Cytidine Deaminase) [7], which preferentially acts on the single-stranded (ss)DNA displaced by DNA:RNA hybrids [9]. Thus, histone mutations were selected only if they increased the appearance of recombinants after inducing AID overexpression, as assayed with the pLZGAID plasmid (**Fig. 1A**, [7]) that contains both the L-*lacZ* direct-repeat recombination system, consisting of two truncated direct repeats of the *LEU2* gene with the bacterial *lacZ* gene placed in-between [10], and the AID cDNA under the control of the GAL promoter [9]. In galactose media, recombinational repair of AID-induced DNA breaks occurring between the repeats by Single-Strand Annealing (SSA) led to deletion of the *lacZ* sequence and formation of a wild-type *LEU2* allele, detectable as Leu+ recombinant colonies (**Fig. 1A**).

However, further experiments showed that some of the mutations enhanced the appearance of recombinant colonies not only after AID overexpression (galactose media) as the selection criteria (**Fig. 1A**), but also under conditions in which AID was not overexpressed (glucose media). These mutations were substitutions of the histone H3 glutamate 73 to glutamine (H3E73Q) or arginine 49 to alanine (H3R49A) and substitutions of the histone histone H4 glutamate 53 to alanine (H4E53A) or glycine 7 to alanine (H4G7A).

In a second phase of the screening, we studied the median frequency of recombination of random colonies from independent transformants with the L, L-*lacZ* and LYΔNS direct-repeat recombination plasmid systems, which differ in the intervening sequence (30 bp, 3 Kb and 5.6 Kb long, respectively) [10,11]. As shown in **Fig. 1B**, only H3E73Q and H4E53A mutants led to a significant increase in the recombination frequencies in all recombination systems and therefore, we proceeded with these two candidates. The increase was further confirmed with the L-*lacZ* system in both mutants. As shown in **Fig. 1C**, H3E73Q and H4E53A mutants led to a significant 8.9- and 63-fold increase in recombination frequencies with respect to the isogenic H3 and H4 wild-type strains, respectively. Thus, H3E73Q and H4E53A mutations increase the levels of direct-repeat recombination regardless of AID, suggesting higher levels of spontaneous DNA breaks that are not associated with DNA:RNA hybrids.

### H3E73Q and H4E53A mutations increase spontaneous recombinogenic DNA damage

**Figure 2:**
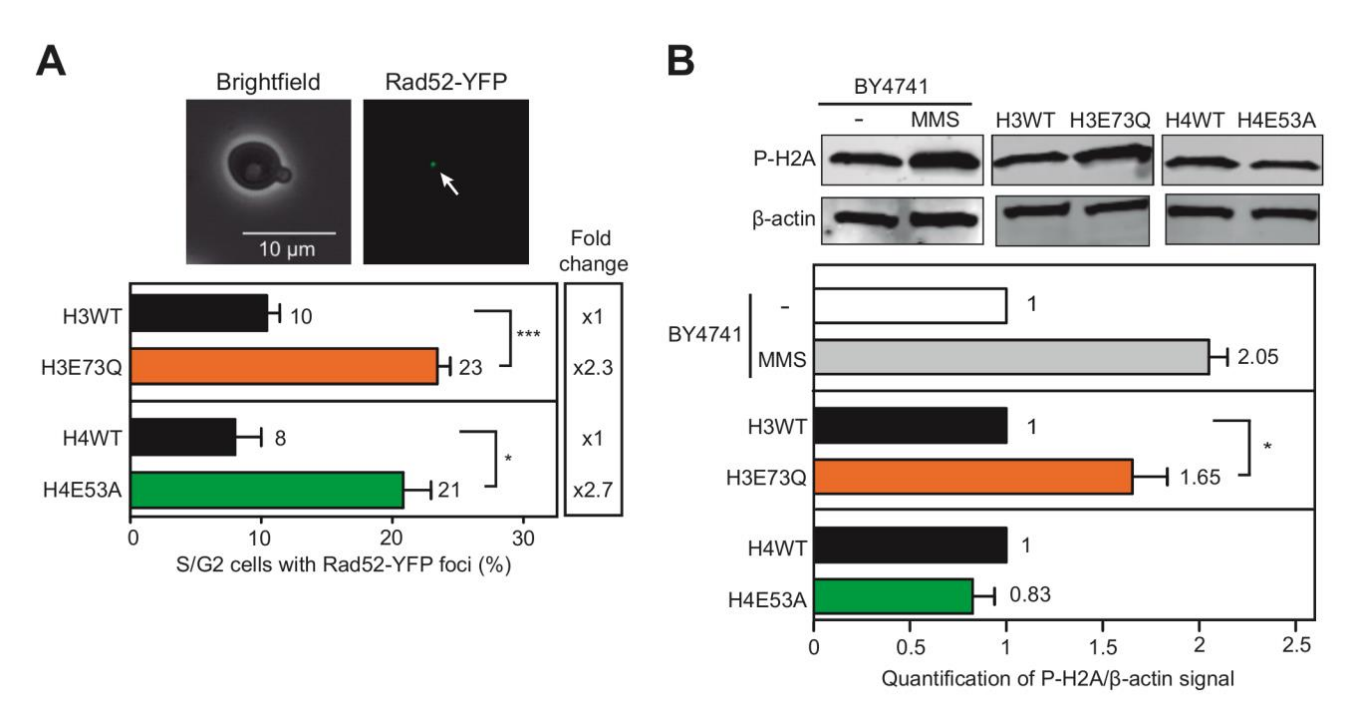
FIGURE 2: Histone H3E73Q or H4E53A mutants increase DNA damage. **(A)** Analysis of the percentage of S/G2 cells containing Rad52-YFP foci in H3 wild-type (H3WT), H3E73Q (H3E73Q)*,* H4 wild-type (H4WT) and H4E53A (H4E53A) strains transformed with pWJ1344. A representative image of a cell with a Rad52-YFP focus is shown (n = 3). **(B)** Accumulation and quantification of the immunofluorescence signal of P-H2A in H3 wild-type (H3WT), H3E73Q (H3E73Q)*,* H4 wild-type (H4WT) and H4E53A (H4E53A) strains detected by fluorescence-based western blot (n = 3). A representative fluorescence-based western blot and the BY4741 strain treated with 0.05% MMS for 1.5 hours as a control (n = 2) is shown. Actin is shown as the loading control. Means and SEM are plotted in all panels. *p ≤ 0.05, ***p ≤ 0.001 (two-tailed Student's t-test).

To measure spontaneous DNA damage, we determined the levels of Rad52-YFP foci, indicative of repair centers that appear in the S and G2 phases of the cell cycle concurring with the coordination between recombination and replication [12]. As shown in **Fig. 2A**, both H4E53A and H3E73Q mutants significantly enhanced the number of cells with Rad52-YFP foci (2.3- and 2.7-fold respectively) thus indicating that H3E73Q and H4E53A mutations induce recombinogenic damage. We also analyzed the levels of P-H2A as a marker of DSBs [5] (**Fig. 2B**). As a control, we also tested P-H2A in the parental wild-type strain (BY4741) and after treatment with 0.05% methyl methane-sulfonate (MMS) during 1.5 hours, which led to a 2-fold increase. Whereas H3E73Q also led to a significant increase of P-H2A, H4E53A showed no differences with the wild-type. Thus, although both H3E73Q and H4E53A mutants accumulate recombinogenic DNA damage, only H3E73Q led to a detectable increase in phosphorylated H2A.

### The hyper-recombination of H4E53A and H3E73Q mutants does not depend on transcription

**Figure 3:**
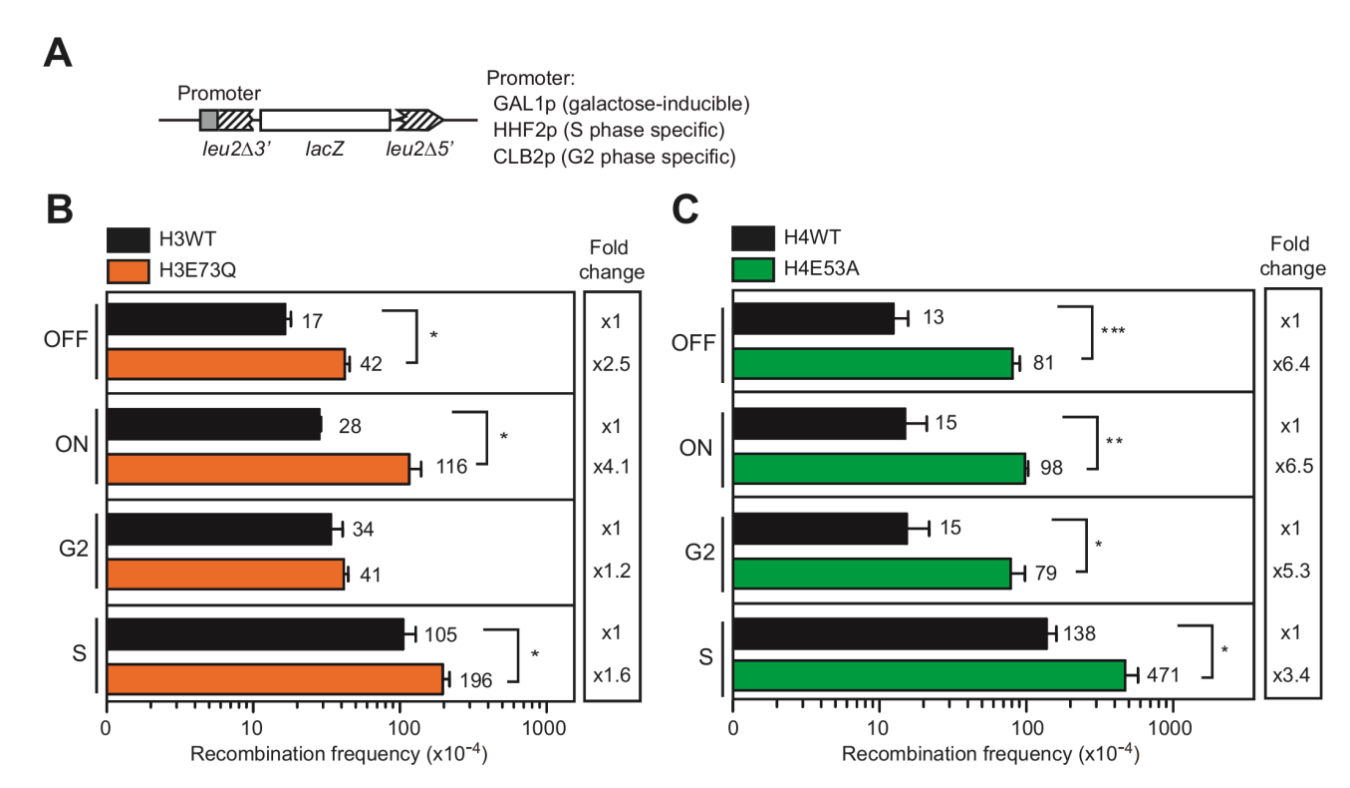
FIGURE 3: Hyper-recombination phenotype of H3E73Q and H4E53A mutants is not associated with defective transcription. **(A)** A scheme of the direct-repeat recombination system with different promoters is shown. *GAL1* promoter (*GAL1p*) is induced in 2% galactose (ON) and repressed in 2% glucose (OFF). Transcription from *CLB2* promoter (CLB2p) is restricted to G2 phase. Transcription from *HHF2* promoter (HHF2p) is restricted to S phase. **(B)** Analysis of direct-repeat recombination frequencies in H3 wild-type (H3WT) and H3E73Q (H3E73Q) strains transformed with pARSLlacZGLB-in, pARSLlacZHLB-IN or pARSLlacZBLB plasmids with the direct-repeat recombination system under *GAL*1, *HHF2, CLB2* promoter respectively (n=3). **(C)** Analysis of direct-repeat recombination frequencies in H4 wild-type (H4WT) and H4E53A (H4E53A) strains transformed with pARSLlacZGLB-in, pARSLlacZHLB-IN or pARSLlacZBLB plasmids with the direct-repeat recombination system under *GAL1, HHF2, CLB2* promoters, respectively (n=3). Means and SEM are plotted in (B) and (C). *p ≤ 0.05, **p ≤ 0.01, ***p ≤ 0.001 (two-tailed Student's t-test).

Given that transcription is a major source of spontaneous DNA damage in the cell, we wondered whether the increase of recombinogenic DNA damage observed in H3E73Q and H4E53A mutants was associated with transcription. We used three direct-repeat recombination systems based on the previously described *L-lacZ* system, where the *LEU2* promoter (*LEU2p*) was replaced by either the *GAL1* inducible promoter (*GAL1p*) or the cell cycle-specific promoters (**Fig. 3A**) [13], *HHF2p* or *CLB2p*, which specifically activate transcription at the S or G2 phases, respectively [14, 15]. As shown in **Fig. 3B** and **3C**, increased levels of recombination were obtained in H3E73Q and H4E53A mutants even when transcription was switched off (*GAL1p* in glucose) indicating that transcription has no major role in the hyper-recombination observed. H3E73Q hyper-recombination was further enhanced when transcription was switched on (galactose) (**Fig. 3B**), suggesting that transcription might explain part of the hyper-recombination phenotype of this mutant. Interestingly, recombination was only enhanced by transcription in the S phase, but not in the G2 phase (**Fig. 3B**), suggesting that transcription-replication conflicts may contribute to the damage observed in this mutant. Instead, recombination was not stimulated by transcription in the H4E53A mutant, since the fold increase in recombination with respect to the wild-type was similar in glucose and in galactose (**Fig. 3C**). Moreover, a significant difference in the levels of recombinants was observed between the wild-type and the H4E53A mutant independent of the moment of the cell cycle in which the recombination system was transcribed (**Fig. 3C**) arguing against transcription as a source of DNA damage in this mutant. Importantly, the fact that both H3E73Q and H4E53A mutants led to increased levels of recombination when transcription was switched off indicates that the main source of recombinogenic DNA damage in these mutants does not depend on transcription.

### H3E73Q and H4E53A mutants increase DNA damage during replication

**Figure 4:**
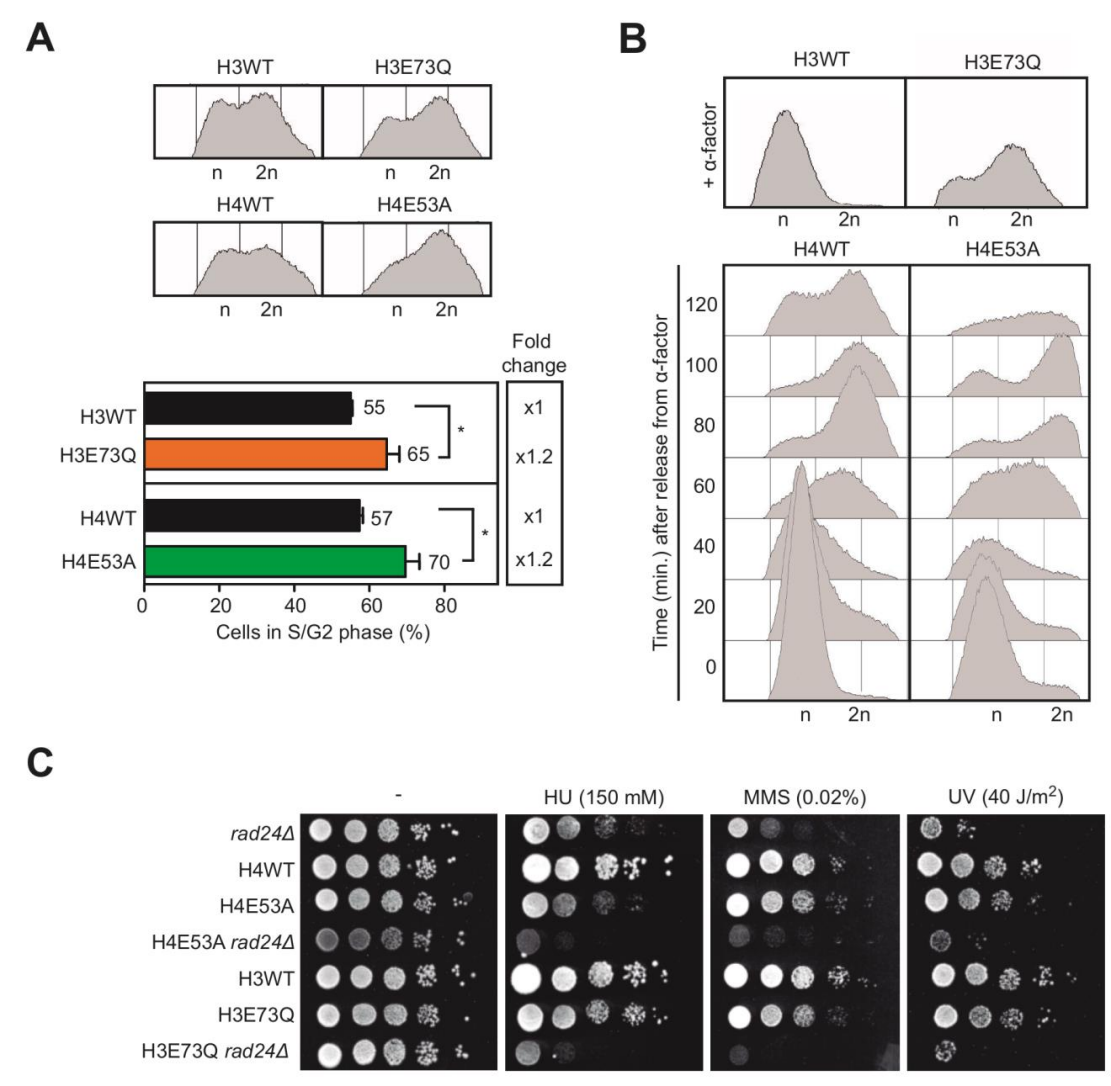
FIGURE 4: Histone H3E73Q and H4E53A mutants increase replication stress. **(A)** Analysis and quantification of cell cycle phases in asynchronous cultures of H3 wild-type (H3WT), H3E73Q (H3E73Q)*,* H4 wild-type (H4WT) and H4E53A (H4E53A) strains by FACS (n=3). **(B)** Analysis of cell cycle progression in H3 wild-type (H3WT), H3E73Q (H3E73Q)*,* H4 wild-type (H4WT) and H4E53A (H4E53A) strains by FACS. **(C)** Sensitivity to HU (150 mM), MMS (0.02%), UV (40 J/m^2^) of *rad24*Δ (R24), H3 wild-type (H3WTn), H3E73Q (H3E73Qn)*,* H3E73Q *rad24*Δ (E73QR24), H4 wild-type (H4WTn), H4E53A (H4E53An) and H3E73Q *rad24*Δ (E53AR24) strains coming from a crossing H3E73Q and H4E53A with W303-1B *rad24*Δ. Similar results were obtained with different spores from the same genetic cross. Means and SEM are plotted in (A). *p ≤ 0.05. (two-tailed Student's t-test).

Given that the S phase is when the DNA is most vulnerable during the cell cycle, we next wondered if the hyper-recombination phenotype observed could be a consequence of DNA damage originated during DNA replication. Thus, we analyzed by FACS the distribution of cells during the cell cycle. We noticed that cells of both H3E73Q and H4E54A mutants accumulate in the S/G2 phase, suggesting DNA replication problems (**Fig. 4A**). We then decided to study cell cycle progression after synchronization in G1 with α-factor. H4E53A cells presented a delay in cell cycle progression though the S phase clearly detectable between 60 and 100 minutes after G1 release (**Fig. 4B**). However, H3E73Q mutants did not respond to α-factor and could not be included in the analysis (**Fig. 4B**).

If H3E73Q and H4E54A mutations were affecting replication, we reasoned that challenging these mutants with genotoxic agents that generate damage during the S/G2 phase would affect their growth. To test that idea, we analyzed growth in media containing either hydroxyurea (HU), which depletes the dNTP pools, or MMS, a DNA alkylating agent. In addition, we tested sensitivity to ultraviolet light (UV), which causes DNA damage throughout the cell cycle. In the single histone mutants, we did not detect large effects in cell viability with any of the treatments suggesting that the amount of DNA damage generated was low and efficiently counteracted by the repair systems (**Fig. 4C**). Thus, given that the checkpoint machinery is required for cell survival upon DNA damage, we decided to further challenge these histone mutants and study the genetic interaction with mutations in the DNA damage checkpoint, a strategy previously used to reveal the role of the checkpoint in transcription-associated DNA damage [16]. For this, we generated double mutants of H3E73Q and H4E54A with the *rad24*Δ checkpoint mutant and analyzed the growth after HU, MMS or UV exposure. As shown in **Fig. 4C**, *rad24*Δ impaired survival of H4E53A or H3E73Q to all the genotoxic agent treatments. This argues that both H4E53A and H3E73Q mutants accumulate lesions that require the DNA damage checkpoint for survival under further stress. Altogether, these results suggest that hyper-recombination of H3E73Q and H4E53A mutants is the result of DNA damage accumulation likely during replication.

## DISCUSSION

**Figure 5:**
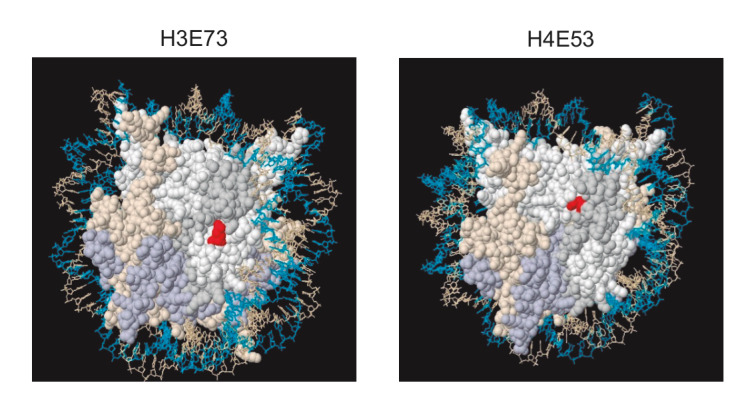
FIGURE 5: Location of H3E73Q and H4E53A mutations. 3D view of the nucleosome crystal structure of the yeast nucleosomes with H3E73 and H4E53 residues colored in red. The images were obtained from www.histonehits.org [18].

In this study, we have identified and characterized two mutations of histone H3 and H4 (H3E73Q and H4E53A) that increase genome instability. H3E73Q and H4E53A residues are conserved and located on the disk surface of the nucleosome thus not interacting with DNA (**Fig. 5**). Both mutations caused hyper-recombination (higher in H4E53A) and increased spontaneous DNA damage. Although evidence from our and other labs in the last two decades has shown that transcription is a major cause of genome instability [17], we show that hyper-recombination is independent of in H3E73Q and H4E53A (**Fig. 3**). Both, H3E73Q and H4E53A mutations increased the percentage of cells with Rad52 foci, cells in the S/G2 phases, and sensitivity to genotoxic agents when the DNA damage checkpoint was inactivated by deleting *RAD24*. Altogether, the data suggests that these two histone residues have a role in preventing recombinogenic DNA damage during the S phase.

Interestingly, H3E73Q and H4E53A also conferred specific phenotypes. Thus, only H3E73Q, but not H4E53A led to increased P-H2A levels, likely reflecting the accumulation of spontaneous DSBs. There was not a hyper-recombination phenotype in H3E73Q when the promoter was active in G2 but since a 2.5-fold recombination increase was still observed when switching off transcription (*GAL1p* in glucose), it seems that transcription is not the major driver of DNA damage in neither of the mutants. The fact that H3E73Q cells could not be synchronized with α-factor might be due to loss of heterochromatin silencing since the H3E73 residue lies in the LRS (Loss of rDNA Silencing) domain. This domain is necessary for sirtuins to deacetylate heterochromatin keeping it silenced [18–20] and when the *HML* and *HMR* heterochromatic regions are expressed, haploid yeasts are not able to respond to α-factor [21]. Indeed, H3K73Q has been reported to affect *HMR* and telomeric silencing [18]. However, this effect is not complete and indeed, we were able to cross this mutant to generate double mutants with *rad24*Δ (**Fig. 4C**). Furthermore, it is also possible that this partial suppression of silencing is involved in the hyper-recombination conferred by H3E73Q since heterochromatin de-silencing can alter replication timing, that has been previously associated with genome instability [22, 23]. Similarly, the increase of DSBs (**Fig. 2B**), S/G2 cells containing Rad52 foci (**Fig. 2A**) and cells in the S/G2 phase observed in H3E73Q mutant (**Fig. 4A**) could also be due to the reported defective silencing of telomeric regions [18]. These observations will explain the DNA damage sensitivity observed when we deleted *RAD24* in H3E73Q mutant. However, provided the potential pleotropic effect of histone modifications in silencing, gene expression and DNA transactions, further detailed analysis would be necessary to define the specific molecular mechanisms by which the H3E73Q mutation compromises genome integrity.

By contrast, the H4E53A mutant responds to α-factor and shows a delay in S phase progression (**Fig. 4B**), which supports that DNA damage accumulates during replication. H4E53A might be directly or indirectly affecting the replication process itself, in this case clearly in a transcription-independent manner (**Fig. 3C**). Interestingly, the H4E53 residue has been reported to interact with Cac1, the largest subunit of the Chromatin Assembly Factor 1 (CAF-1), which, together with Asf1, promotes histone H3 and H4 deposition onto newly synthesized DNA during replication, what is essential for proper S-phase progression [24, 25]. Thus, it is tempting to speculate that H4E53A might impair the interaction with Cac1 affecting S-phase progression and leading to the observed DNA damage accumulation. However, again further detailed analysis would be necessary to define the specific molecular mechanisms by which this mutation compromises genome integrity.

In summary, our study uncovered a key function of the conserved H3E73 and H4E53 histone residues in the maintenance of genome integrity by preventing the formation of recombinogenic DNA damage, adding new light into our understanding of the role of histones in the mechanisms of genome integrity that would need to be explored further.

## MATERIALS AND METHODS

### Yeast strains and media

BY4741, H3WT, H4WT, H3E73Q, H3R49A, H4E53A and H4G7A yeast strains have been reported previously [7, 8]. H3E73Q and H4E53A were crossed with W303-1B *rad24*Δ (WR24-6C) [16] to obtain *hht1-hhf1::NatMX4 hht2-hhf2::[H3]-URA3* (H3WTn), *hht1-hhf1::NatMX4 hht2-hhf2::[H4]-URA3* (H4WTn), *TRP1 hht1-hhf1::natMX4 hht2-hhf2::[E73Q]-URA3* (H3E73Qn) and *hht1-hhf1:: natMX4 hht2-hhf2::[E53A]-URA3* (H4E53An), *rad24*Δ*::TRP1 hht1-hhf1:: natMX4* (R24), *rad24*Δ*::TRP1 hht1-hhf1::natMX4 hht2-hhf2::[E73Q]-URA3* (E73QR24) and *rad24*Δ*::TRP1 hht1-hhf1:: natMX4 hht2-hhf2::[E53A]-URA3* (E53AR24).

Media used in this study: YPAD (1% yeast extract, 2% bacto-peptone, 2% glucose, 20 mg/L adenine), SD (0.17% yeast nitrogen base (YNB) without amino acids nor ammonium sulfate, 0.5% ammonium sulfate and supplemented with amino acids. The absence of amino acid/s is specified when required), SC (SD containing 2% glucose) and SPO (1% potassium acetate, 0.1% yeast extract, 0.005% glucose). Solid media were prepared adding 2% agar before autoclaving.

Yeast strains were freshly defrosted from stocks and grown at 30°C. All experiments were performed at 30°C.

### Plasmids

All plasmids used in this study were previously reported. pWJ1344 contains the Rad52::YFP construct [12]. pRS316-L is a centromeric plasmid containing a *leu2*Δ*3′::leu2*Δ*5′* direct-repeat construct [11]. pRS314L*lac*Z is a centromeric plasmid containing the *leu2*Δ*3′::leu2*Δ*5′* direct-repeat construct with the *lacZ* gene in between the repeats [10]. pRS316-LYΔNS is a centromeric plasmid containing the *leu2*Δ*3′::leu2*Δ*5′* direct-repeat construct with a fragment of the Ylp5 plasmid in between the repeats [11]. pLZGAID is a centromeric plasmid containing containing the *LlacZ* construct and the AID under the *GAL1* promoter [7]. pARSLl*acZ*GLB-IN, pARSLl*acZ*HLB-IN and pARSLl*acZ*BLB-IN are centromeric plasmids containing the *LlacZ* construct transcribed from the *GAL1, HHF2* and *CLB2* promoters, respectively [13, 26].

### Genetic analysis of recombination

For the recombination assays with the direct-repeat systems, cells were grown on plates with SC lacking tryptophan for 3 to 4 days. Recombinants were selected on plates with SC medium lacking leucine. In Fig. 1A, qualitative recombination frequencies of different mutants from the collection of non-essential histone H3 and H4 mutants were obtained. In Fig. 1B recombination frequencies were the median value of a total of at least eleven colonies coming from three independent transformants. In Fig. 1C and 3, recombination frequencies were obtained by fluctuation tests as the median value of six independent colonies isolated from plates with SC medium. The final frequency given for each strain and condition is the mean and SEM deviation of three to four median values, as described previously [27].

### Analyses of Rad52-YFP foci

Rad52-YFP foci were counted in more than 200 S/G2 cells transformed with pWJ1344. Cells were visualized in Leica DC 350F. The mean and SEM of three different experiments were plotted.

### Western blot analysis

10 mL of each strain culture at 0.7 (O.D. 600 nm) growing in SC were recovered and kept on ice. The culture was centrifuged and proteins were extracted from pellets by adding 200 μL of cold 10% TCA and 200 μL of glass beads by vortexing 7 times during 20 seconds each time at 4°C. Supernatant was recovered and beads were washed twice with 200 μL of cold 10% TCA. Samples were centrifuged 10 minutes at 3000 rpm and supernatants were discarded. The remaining pellet was resuspended using 100 μL of loading Buffer (62.5 mM Tris-HCl pH 6.8, 25% glycerol, 2% SDS, 0.01% water-dilluted Bromophenol Blue, 5% β-mercaptoethanol), 50 μL of water and 50 μL of 1M Tris (not-adjusted pH). Prior to gel loading samples were boiled for 5 minutes and centrifuged 10 minutes at 3000 rpm at room temperature. Proteins were separated on BIO-RAD mini-Protean TGX Gel and wet-transferred to PVDF membranes (Immobilon-FL, Milipore). Antibody 8332 (Abcam, UK) for β-actin was used at a 1:1000 and antibody 39271 (BIOMOL) for P-H2A was used at 1:2000 dilution, both in 1x TBS-0,1%-Tween and incubated 1 hour with fluorescence secondary antibody IRDW 600CW or 800 CW for signal detection. The actin signal is shown as a loading control and its levels were used to normalize the amount of P-H2A for each transformant. The mean and SEM of three different experiments were plotted.

### Cell cycle synchronization and flow cytometry

Cells were arrested in the G1 phase with 2.5 µM of α-factor mating pheromone. Approximately 1 mL (10^7^ cells) were collected from G1-arrested or asynchronous cultures at the indicated time points centrifuge 1 minute at 5000 rpm and washed twice with sodium citrate 50 mM pH 7.5. Cells were resuspended in 1 mL of sodium citrate 50 mM pH 7.5 and incubated one hour with 25 μL of RNase A (10 mg/mL) and one hour with 20 mg/mL of proteinase K at 50°C. Cells were incubated 1 hour at 50°C after the addition of 1 mL sodium citrate 50 mM pH 7.5 containing 16 μg/mL propidium iodide. Cell cycle distribution was analysed using a FACSCalibur system (Becton-Dickinson).

### Genotoxic-sensitivity assays

Mid-log cultures were grown in YPAD medium. 10-fold dilutions of the culture prepared in sterile water were plated on solid YPAD medium containing the drugs at the indicated concentrations. UV irradiation was performed with the dried plates. Plates were incubated during 3 days (in the dark for UV-irradiated plates).

## AUTHOR CONTRIBUATION

P.O., D. G.-P and M. SM.-A. performed the experiments; P.O., A. R., D. G.-P., B.G.-G., and A.A. designed the experiments. P.O., B.G.-G., and A.A. wrote the manuscript. All authors read, discussed, and agreed with the final version of this manuscript.

## Abbreviations:

AID: – Activation-Induced Cytidine Deaminase,
DSB: – double-strand break,
HU: – hydroxyurea,
MMS: – methyl methane-sulfonate,
UV: – ultra violet.
